# Detection and Characterization of β-Lactam Resistance in *Bacillus cereus* PTCC 1015

**DOI:** 10.1100/tsw.2004.122

**Published:** 2004-08-12

**Authors:** Javad Behravan, Zeinab Rangsaaz

**Affiliations:** Pharmaceutical Biotechnology Research Center, Mashhad University of Medical Sciences, Mashhad, IRAN; ^2^ Biotechnology Section, School of Pharmacy, Mashhad University of Medical Sciences, Mashhad, IRAN

**Keywords:** ampicillin, Bacillus cereus, β-lactamase, cephalexin

## Abstract

In the present study, detection, isolation, and characterization of β-lactamases from *Bacillus cereus* PTCC 1015 were investigated. *B. cereus* was inoculated in nutrient broth containing ampicillin (50 μg.ml^−1^) for 24 h (35°C, 200 rpm). Activity measurements were carried out against ampicillin (0.1 mg.ml^−1^) and cephalexin (0.08 mg.ml^−1^) by a spectrophotometric method at different conditions (pH 6–10, temperatures 25—45°C).Maximum penicillinase and cephalosporinase activity was observed at pH 7. The optimized temperatures for penicillinase and cephalosporinase activity were 30 and 40°C, respectively. At the above conditions, maximum enzymatic activity was calculated as 0.89 ± 0.014 and 0.037 ± 0.001 units against ampicillin and cephalexin.

## INTRODUCTION

The widespread use of antibiotics has put bacteria under tremendous selective pressure to devise mechanisms to escape the lethal action of the drugs. The increase in resistance to β-lactam antibiotics and the broad activity spectrum of β-lactamases in many pathogenic bacteria are frightening. In pathogenic bacteria, β-lactamase production is the most important contributing factor to β-lactam resistance[1]. β-Lactamases catalyze the hydrolysis of an amide bond in the β-lactam ring of penicillins and caphalosporins, rendering a species that is no longer an inhibitor of bacterial transpeptidases[2].

On the basis of amino acid sequence similarities, β-lactamases have been divided into four classes, A to D. Class A, C, and D enzymes are serine hydrolyzes while class B contains metallo-β-lactamases. The three classes of serine β-lactamases are evolutionarily related and do not share any sequence or structure similarity with zinc-β-lactamases[3]. In total, at least 340 β-lactamases with unique amino acid sequences or differentiated phenotypic behavior have been isolated from clinical isolates[4]. Due to their efficient enzymatic activity, β-lactamases have been used in development of sensitive and reliable reporters for gene expression in intact mammalian tissue culture cells and zebrafish embryos[5,6].

*Bacillus cereus* strain 569/H exhibits resistance to penicillin and other β-lactam agents. Three different β-lactamases, named β-lactamase I, II, and III, have been reported for this strain[7]. According to the classification scheme proposed by Bush et al., *B. cereus* β-lactamases I and III are group A enzymes, while the β-lactamase II is a heat-stable metallo-β-lactamase[3].

Here we report the isolation and β-lactamase activity of *B. cereus* PTCC 1015. To the best of our knowledge, its β-lactamase activities have not been studied before. The study was conducted using various conditions of pH and temperature against two substrates, ampicillin and cephalexin.

## MATERIALS AND METHODS

- *Maintenance and preservation of B. cereus*. Cultures were maintained on sterile nutrient agar slants. The strain was grown at 37°C. Plates containing spore-bearing colonies were stored at room temperature for up to 1 month. Long-term storage of the strain was at –20°C in 20% glycerol in nutrient broth.
- *Susceptibility testing.* Antibiotic susceptibilities were determined by standard disc diffusion[8]. Susceptibilities to the following β-lactam antibiotics were determined: amoxicillin, ampicillin, cefazolin, ceftizoxim, ceftriaxon, cephalexin, cloxacillin, and penicillin. *Staphylococcus aureus* ATCC 29737 was used simultaneously for control purposes.
- *Production of β-lactamases. B. cereus* PTCC 1015 was grown in shake flasks containing 100 ml nutrient broth (at 200 rpm, 35°C for 24 h). β-Lactamase production was induced with 0.1 $mg.ml^{-1}$ of ampicillin or cephalexin where appropriate.
- *Assay for β-lactamase activity.* β-Lactamase activity was determined by a spectrophotometric method[9,10], measuring the decrease in absorbance at an appropriate wavelength. The wavelengths used (257 nm for ampicillin and 260 nm for cephalexin) were those that gave maximums in a difference spectrum when a nonhydrolyzed substrate was scanned against a hydrolyzed one. The unit of β-lactamase activity assayed is the amount of enzyme that hydrolyzes 1 μmol substrate per minute at 30°C and pH 7.
- *Effects of temperature and pH on β-lactamase activity*. Thermal stability and optimum temperature of β-lactamase activity were determined between 25–45°C. The enzyme activity was also assayed between pH values of 6–10.
- *Sterilization and aseptic techniques.* All culture media were sterilized by autoclaving at a steam pressure of 103.5 kPa (15 $lb.in^{-2}$), corresponding to a temperature of 121°C, for 15 min. All inoculum preparations and culture transfers were carried out under aseptic conditions.

## RESULTS

All results presented in this paper are the means of three replicate assays. The antimicrobial activity profiles for the various β-lactam antibiotics against *B. cereus* and *S. aureus* are presented in Table 1. In all cases, the *B. cereus* strain characteristically showed no sensitivity or resistance compared to the control strain.

During the exponential phase of growth in which the analytic measurements were made, the β-lactamase activity in the medium increased rapidly, but the rates of increase declined as the culture approached its stationary phase. The effect of pH on β-lactamase activity of the microorganism at 25°C was determined in the absence of inducers. Maximum activity against ampicillin (2 $mg.ml^{-1}$) and cephalexin (0.08 $mg.ml^{-1}$) was observed at pH 8 (Table 2 and Fig. 1).

**TABLE 1**
**Inhibition Zone Diameter of Some β-Lactam Antibiotics* for *B. cereus* PTCC 1015 and *S. aureus* ATCC 29737 Obtained by Disc Diffusion Method**

| | Mean Zone Diameter, mm | | | | | | | |
|---|---|---|---|---|---|---|---|---|
| | CLOX | CEPH | CEFR | PCIN | AMPI | CEFT | AMOX | CEFA |
| *S. aureus* | 0 | 29 | 16 | 23 | 26 | 19 | 27 | 27 |
| *B. cereus* | 0 | 0 | 0 | 0 | 0 | 0 | 0 | 10 |

* Abbreviations: CLOX, cloxacillin; CEPH, cephalexin; CEFR, ceftriaxon; PCIN, penicillin; AMPI, ampicillin; CEFT, ceftizoxim; AMOX, amoxicillin; CEFA, cefazolin.

**TABLE 2**
**Effects of pH on β-Lactamase Activity* of the Crude Extract Against Ampicillin (2 mg.ml$^{-1}$) and Cephalexin (0.08 mg.ml$^{-1}$) at 25$^{o}$C**

| | Ampicillin | | | | Cephalexin | | | |
|---|---|---|---|---|---|---|---|---|
| pH | A1 | A2 | A3 | A±SD | A1 | A2 | A3 | A±SD |
| 6 | 0.255 | 0.257 | 0.248 | 0.253 ± 0.005 | 0.016 | 0.014 | 0.015 | 0.015 ± 0.001 |
| 7 | 0.467 | 0.490 | 0.480 | 0.479 ± 0.011 | 0.011 | 0.011 | 0.013 | 0.012 ± 0.001 |
| 8 | 0.521 | 0.530 | 0.529 | 0.527 ± 0.005 | 0.023 | 0.026 | 0.024 | 0.024 ± 0.001 |
| 9 | 0.504 | 0.525 | 0.507 | 0.512 ± 0.011 | 0.020 | 0.018 | 0.020 | 0.019 ± 0.001 |
| 10 | 0.304 | 0.329 | 0.315 | 0.316 ± 0.012 | 0.017 | 0.016 | 0.014 | 0.016 ± 0.001 |

***** β-Lactamase activity is expressed as the amount of the extract (mL) which hydrolyzed 1 µmol substrate per minute at 25$^{o}$C at the respective pH.

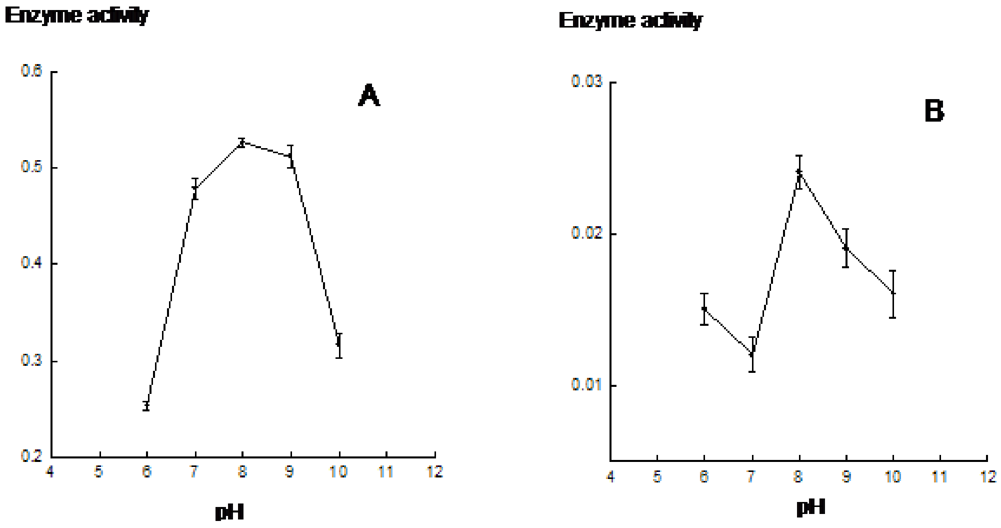

**FIGURE 1.** Spectrophotometric evaluation of the extracts β-lactamase activity (not induced) against ampicillin (A) and cephalexin (B) at 25$^{o}$C.

The pH value was then kept unchanged and the β-lactamase activity of crude extract was evaluated at temperatures 25–45°C against ampicillin and cephalexin. Maximum β-lactamase activity against ampicillin was found to be at 40°C while highest activity against cephalexin was observed at 30–40°C (Fig. 2). Similar experiments using inducers were carried out. In the presence of inducers, maximum β-lactamase activity against ampicillin and cephalexin was determined. The highest measurable β-lactamase activity against ampicillin in our experiments was found at pH 6 and at 30°C, while the optimum conditions for β-lactamase activity against cephalexin was found to be at pH 8 and 40°C (Fig. 3 and Fig. 4).

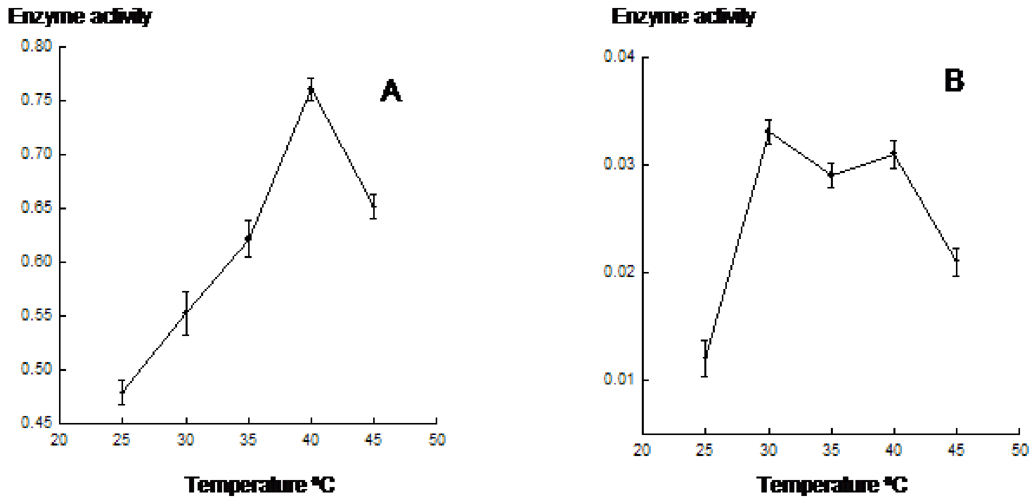

**FIGURE 2.** Spectrophotometric evaluation of the extract β-lactamase activity (not induced) against ampicillin (A) and cephalexin (B) at pH 7.

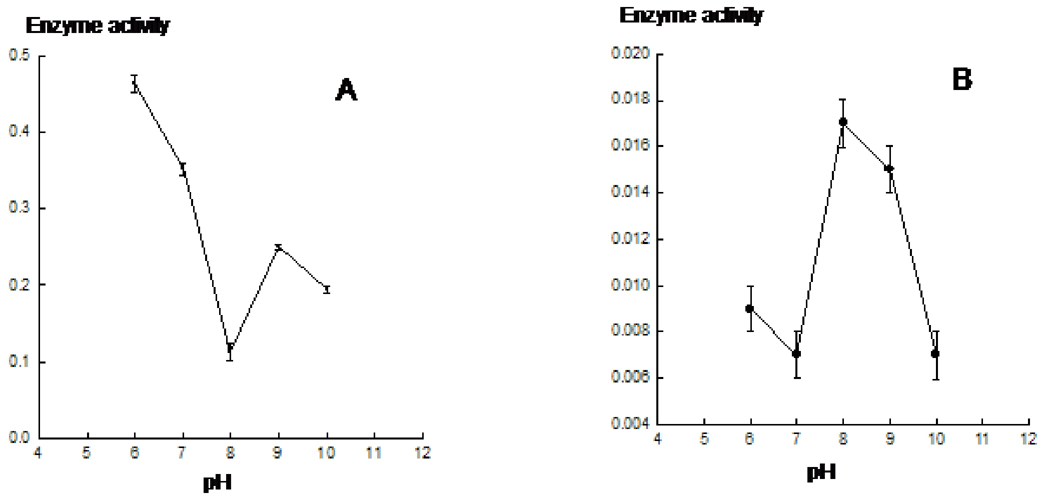

**FIGURE 3.** Spectrophotometric evaluation of the extracts β-lactamase activity (induced) against ampicillin (A) and cephalexin (B) at 25°C.

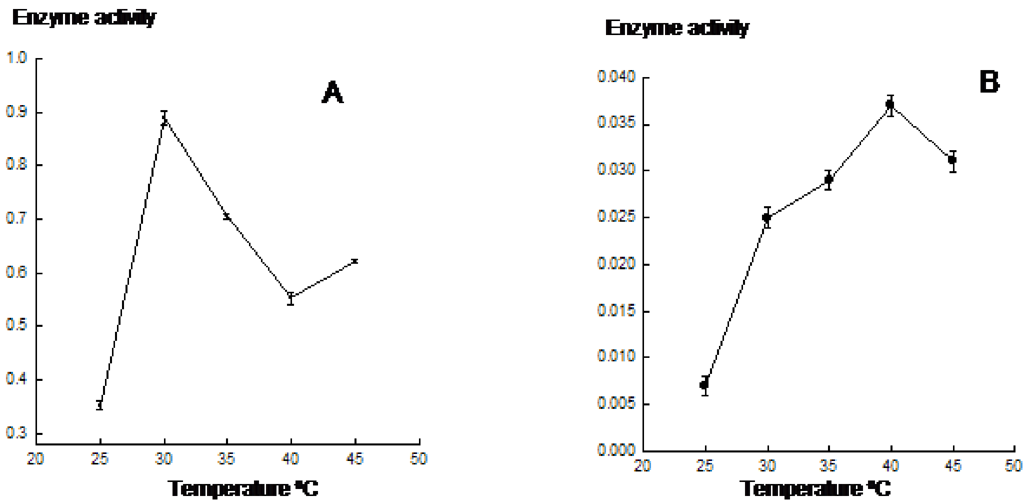

**FIGURE 4.** Spectrophotometric evaluation of the extract β-lactamase activity (induced) against ampicillin (A) and cephalexin (B) at pH 7.

## DISCUSSION AND CONCLUSIONS

*B. cereus* strains commonly exhibit resistance to penicillin and other β-lactam agents. Three distinct β-lactamases have been characterized for *B. cereus* 569/H in which these enzymes described to be inducible[7]. In this project, the β-lactamase activity of another unstudied strain, *B. cereus* PTCC 1015 was evaluated against ampicillin and cephalexin using different assay conditions[11]. When the production of β-lactamases was not induced, optimal pH for the penicillinase and cephalosporinase activity was found to be 8. When the production of β-lactamases was induced, maximum penicillinase activity was observed at pH 6 and 30°C, while the highest cephalosporinase activity was determined to be at pH 8 and 40°C. These results are closely related to the results reported by Berks et al.[12], who observed that maximum activity for β-lactamase (cephalosporinase) in *Pseudomonas aeruginosa* NCTC 8203 was at pH 8.5 and different from those reported by Livermore and Corkill[13], who found that pH values 6–7 were optimal for β-lactamase activity in *Escherichia coli* 976 (TEM-1 type β-lactamase). This could be explained by the fact that properties of different β-lactamases vary extremely, and it is important to study them in each strain[3].

The effect of temperature on the β-lactamase activity is shown in Fig. 2. Optimum temperature was 30–40°C. Similar behavior has been reported by Saino et al.[14]. The constitutive and inducible β-lactamases are closely similar in their catalytic activities. It appears that the synthesis of these two groups of enzymes by the same microorganism is mediated by two different structural genes.

More investigations are underway to obtain a complete characterization of the β-lactamases, determining their kinetic properties and behavior with respect to different physical and chemical agents.

## ACKNOWLEDGMENTS

This work was supported by a grant from the research committee, School of Pharmacy, Mashhad University of Medical Sciences, Iran.
